# From cool to non-cool: California’s ‘non-menthol’ cigarettes in 2025

**DOI:** 10.18332/tpc/217322

**Published:** 2026-05-27

**Authors:** Sairam V. Jabba, Hanno C. Erythropel, Paul T. Anastas, Stephanie S. O’Malley, Suchitra Krishnan-Sarin, Julie B. Zimmerman, Sven E. Jordt

**Affiliations:** 1 Department of Anesthesiology, Duke University School of Medicine, Durham, United States; 2 Yale Tobacco Center of Regulatory Science, Department of Psychiatry, Yale School of Medicine, New Haven, United States; 3 Department of Chemical and Environmental Engineering, Yale University, New Haven, United States; 4 Center for Green Chemistry and Green Engineering, Yale School of the Environment, New Haven, United States; 5 School of Public Health, Yale School of Medicine, New Haven, United States; 6 Department of Psychiatry, Yale School of Medicine, New Haven, United States

**Keywords:** regulation, menthol cigarettes, WS-3, cooling agents, characterizing flavors

## Abstract

Since late 2022, the sale of most flavored tobacco products has been prohibited in California, including menthol cigarettes. Tobacco companies responded by introducing ‘non-menthol’ cigarettes in which menthol was replaced with WS-3, an odorless synthetic cooling agent to elicit cooling sensations similar to menthol. Legislation enacted in 2024 banned the addition of cooling characterizing flavors in tobacco products in California. However, the industry continues to market ‘non-menthol’ cigarettes in the state, with very similar package designs. The aim of this study was to verify whether cooling agents were removed from these cigarettes. Available Newport-branded ‘non-menthol’ cigarettes were purchased in California in 2025, extracted and tested for sensory cooling activity by Ca^2+^ microfluorimetry of HEK293T cells expressing the human TRPM8 cold/menthol receptor. Chemical analysis was performed by gas chromatography - mass spectrometry (GCMS). ‘Non-menthol’ and menthol cigarettes marketed in 2023–2024 served as controls. While extracts from Newport ‘non-menthol’ and menthol cigarettes marketed in California in 2023 produced a TRPM8-mediated Ca^2+^ increase of 60 ± 8% and 39 ± 3%, (p<0.0001, n=3) respectively, responses elicited by extracts of Newport ‘non-menthol’ cigarettes marketed in 2025 were indistinguishable from baseline (p=0.48, n=3). Chemical analysis confirmed no menthol or WS-3 above the level of detection (10 μg/cigarette), and no other major commercial synthetic cooling agents. The tobacco industry removed sensory cooling agents from ‘non-menthol’ cigarettes marketed in California. However, this did not result in the market withdrawal of ‘non-menthol’ cigarettes in the state. ‘Non-menthol’ cigarettes in California continue to be marketed with package designs resembling those of former menthol cigarettes, signaling the potential presence of a characterizing flavor.

## INTRODUCTION

**Figure 1:**
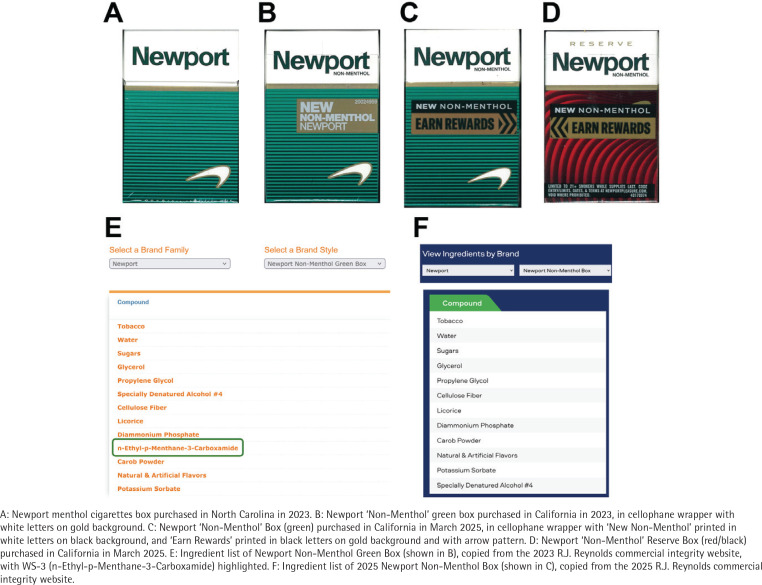
New ‘non-menthol’ cigarettes introduced in California in 2025

Since 21 December 2022, the sale of most flavored tobacco products has been prohibited in California^1^. This ban includes menthol cigarettes that were previously favored by youth and young adults, women and non-Hispanic Black Americans^2^. In the same month, tobacco companies introduced ‘non-menthol’ cigarettes in California, advertising them with designs highly similar to former menthol cigarette brands, including R.J. Reynolds Tobacco’s Newport (Figures 1A and 1B) and Camel and Imperial Tobacco Group (ITG) Brands’ Kool cigarettes^3-5^. Total cigarette sales in California declined by 21.1% in the 18 months after the flavor ban, mostly due to the abrupt decline in menthol cigarette sales^6^. However, this was partially offset by sales of ‘non-menthol’ cigarettes^6^. Some brands of ‘non-menthol’ cigarettes, Newport Green and Camel Crisp, were found to contain WS-3, a synthetic sensory cooling agent^3,4^. California regulators determined that these cigarettes violated the State’s flavor ban; however, R.J. Reynolds filed a judicial complaint stating that WS-3-containing ‘non-menthol’ cigarettes have no characterizing flavors^7^. California lawmakers responded by revising tobacco legislation to include cooling sensations in the definition of characterizing flavors, thereby effectively banning WS-3-containing cigarettes^7,8^.

In March 2025, after California’s new legislation took effect, two new ‘non-menthol’ cigarette varieties were introduced by R.J. Reynolds, including: 1) a Newport variety with altered ‘new non-menthol’ advertising on the wrapper, but otherwise in an unaltered green box; and 2) a Newport Reserve ‘new non-menthol’ variety in a box with red/black wave patterns (Figures 1C and 1D). The company had also changed its ‘Commercial Integrity’ website in which WS-3 was listed as an ingredient in its California-marketed Newport varieties introduced in 2022 (Figure 1E)^9^. On the revised website, WS-3 is not listed as an ingredient of the new varieties introduced in 2025 (Newport non-menthol Box, Newport Reserve non-menthol Box) (Figure 1F). However, the ingredient list includes ‘Natural & Artificial Flavors’ which could include WS-3 or other synthetic cooling agents^10^. The aim of this study was to verify whether sensory cooling agents are indeed absent in these newly introduced ‘non-menthol’ cigarettes.

Newport ‘non-menthol’ cigarettes were purchased in March and May 2025 at convenience stores in the Alameda and San Francisco areas for *in vitro* pharmacological and chemical analysis. Extracts from tobacco rods were serially diluted and superfused over HEK293T cells expressing the human TRPM8 cold/menthol receptor activated by menthol, WS-3 and other synthetic cooling agents, measuring activity by Calcium-microfluorimetry as published (Supplementary file)^3^.

Whole cigarettes (3 cigarettes from 3 different packages of the same variety) were extracted and analyzed in triplicate for the major commercial sensory cooling agents (menthol, WS-3, WS-23, WS-12, Frescolat MGA, Frescolat ML, Frescolat X-cool) by gas chromatography - mass spectrometry following published protocols (Supplementary file)^3^.

## COMMENTARY

**Figure 2:**
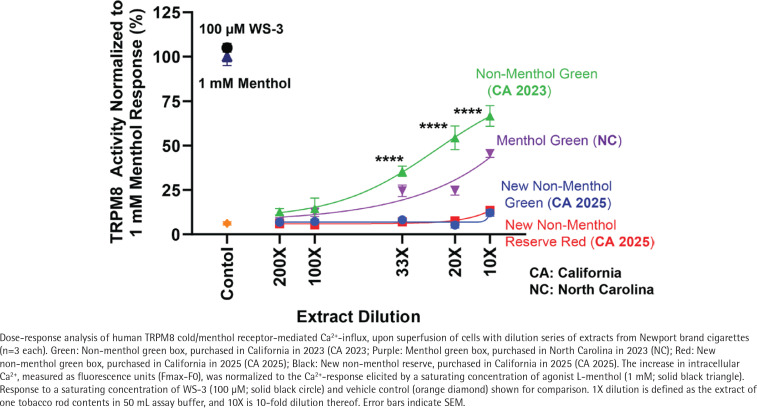
Sensory cooling activity of Newport ‘non-menthol’ cigarettes introduced in California in 2023 and 2025, compared to Newport menthol cigarettes

Extracts from Newport new ‘non-menthol’ (green) cigarettes did not increase intracellular Ca^2+^ levels, even at the lowest dilution tested (10×, p=0.48, n=3), similar to Newport ‘non-menthol’ Reserve (red) unflavored control cigarettes (Figure 2). In contrast, control extracts of 2023 North Carolina-marketed Newport menthol cigarettes and 2023 California-marketed ‘non-menthol’ green cigarettes increased intracellular Ca^2+^ levels in a dose-dependent manner, by 39 ± 3%, (p<0.0001, n=3) and 60 ± 8% (p<0.0001, n=3) due to the previously verified presence of menthol or WS-3 (Figure 2)^3^.

Chemical analysis revealed that none of the Newport ‘non-menthol’ varieties introduced in 2025 contained any of the major commercial cooling agents over the limits of detection, while the WS-3 content of Newport ‘non-menthol’ green cigarettes marketed in 2023 was 1.26 ± 0.05 mg/cig, similar to our previously reported findings^3^.

Taken together, these data demonstrate that R.J. Reynolds removed sensory cooling agents from their California-marketed Newport ‘non-menthol’ varieties. However, this does not mean that ‘non-menthol’ cigarettes have disappeared from California’s market. It is important to note that in the case of the Newport non-menthol green variety (Figure 2C), the box design and color are identical to the previous WS-3-containing version, and very similar to Newport menthol cigarettes (except the ‘non-menthol’ label), which could continue to signal the presence of a minty and cooling characterizing flavor to people who previously smoked menthol cigarettes. R.J. Reynolds’ strategy is similar to ITG Brands’ for their Kool ‘non-menthol’ cigarettes introduced in California in December 2022 that have blue/black or green/black box coloring similar to the brand’s menthol cigarettes, but do not contain any cooling agent^3,4^. A distinctive choice of package coloring and design by the industry is a common strategy to manipulate consumer expectations about the flavor and strength of tobacco products^11^. In June 2023, California regulators raised concerns about the misleading package design and coloring of these Kool ‘non-menthol’ cigarettes^12^. Similar concerns should be raised about the new Newport non-menthol green variety introduced in 2025 that continues to signal that a characterizing flavor could be present. Implementation of plain packaging requirements would likely prevent consumers from assuming the presence of a characterizing flavor based on package design and color^13^.

### Limitations

While highly sensitive, it is possible that the cigarettes tested may contain trace amounts of menthol or cooling agents below the detection limits of our pharmacological and chemical analytical methods. However, such small amounts would not be detected by consumers as characterizing flavors^14^. Sensory evaluations in consumer panels, with and without knowledge of the ‘non-menthol’ descriptor and package color and design, may reveal whether marketing affects consumer perceptions, even in the absence of cooling agents in the products.

## CONCLUSION

The tobacco industry removed sensory cooling agents from ‘non-menthol’ cigarettes marketed in California. However, this did not result in the market withdrawal of ‘non-menthol’ cigarettes in the state. ‘Non-menthol’ cigarettes in California continue to be marketed with package designs resembling those of former menthol cigarettes, signaling the potential presence of a characterizing flavor.

## Supplementary Material



## Data Availability

The data supporting this research are available from the authors on reasonable request.
