# Supplementary material for: From cool to non-cool: California’s ‘non-menthol’ cigarettes in 2025
Source: Tob Prev Cessat. 2026 May 27;12:10.18332/tpc/217322. doi: 10.18332/tpc/217322 (PMC13215131; doi:10.18332/tpc/217322)
Supplement: Supplementary file 1 [file TPC-12-26-s1.pdf]

Supplementary file

Methods

Assessment of sensory cooling activity: Flavour chemicals were extracted from cigarette tobacco rods by stirring the rod material overnight in 10mL methanol. Extracts were strained from solid materials using a nylon strainer and subsequent centrifugation. Supernatants were collected and dried using vacuum centrifugation. Dried constituents were reconstituted in calcium assay buffer (Hank's Balanced Salt Solution with 10 mM HEPES) and further dilutions were prepared (1X-200X) to assess sensory cooling activity by Ca^{2+} microfluorimetry of HEK293t cells expressing the human cold/menthol receptor, TRPM8 (FlexStation, Molecular Devices). 1X dilution is defined as the extract of one tobacco rod contents in 50 mL assay buffer, and 200X is 200-fold dilution thereof). Ca^{2+}-responses from these extracts were normalized to the Ca^{2+}-response elicited by a maximally activating concentration of agonist L-menthol (1 mM; TRPM8).

Chemical analysis: Chemical analysis was carried out as described previously, [3] with slight modification: The tobacco filler was removed and placed in a 20 mL vial, and the wrapping paper and filter material were cut into smaller sections and also added to the same vial. 10 mL of methanol (Fisher Scientific, Waltham, Massachusetts, USA) were added to the vial, and the vials were shaken using an orbital shaker (Thermo Scientific Solaris, Waltham, Massachusetts, USA) at 25 °C for 60 minutes. Extracts were filtered (0.22 µm, Millex-GP, Sigma-Aldrich, St. Louis, Missouri, USA) and 1 µL was injected directly into a gas chromatograph with connected mass spectrometer (GC/MS; Perkin-Elmer Clarus 580-SQ8S, Shelton, Connecticut, USA), which was outfitted with an Elite-5MS column (length 60 m, id 0.25 mm, 0.25 µm film, Perkin-Elmer) using the following heating program: Hold at 30 ºC for 7 min, ramp 10 ºC/min to 50 ºC and hold for 20 min, ramp 10 ºC/min to 310 ºC and hold for 7 min. Commercially available standards

were purchased to confirm presence/absence of the following coolants: Menthol (Supplier: TCI America, Portland, Oregon, USA; LOD: 10 µg/cigarette; LOQ: 30 µg/cigarette); WS-3 (TCI America; 10 µg/cigarette; 30 µg/cigarette); WS-23 (TCI America; 2.5 µg/cigarette; 7.5 µg/cigarette); WS-12 (Taima, Xi'An City, China; 500 µg/cigarette; 1500 µg/cigarette); Frescolat MGA (as l-menthone glycerol ketal, Caldwell, New Jersey, USA; 50 µg/cigarette; 150 µg/cigarette); Frescolat ML (as L-menthyl lactate, Sigma Aldrich; 100 µg/cigarette; 300 µg/cigarette); Frescolat X-Cool (Symrise, Teterboro, New Jersey, USA; 50 µg/cigarette; 150 µg/cigarette).

Statistical analysis: Dose-response curves for receptor activity and associated calcium influx changes were plotted using non-linear regression analysis with a 4-parameter logistic equation (Graphpad Prism 9.0, San Diego, California, USA). Error bars indicate Standard Error of the Mean (SEM).

© **2026 Jabba S.V. et al.**
